# Acetyl Fentanyl Overdose Fatalities — Rhode Island, March–May 2013

**Published:** 2013-08-30

**Authors:** Laurie Ogilvie, Christina Stanley, Lauren Lewis, Molly Boyd, Matthew Lozier

**Affiliations:** Rhode Island State Health Laboratories; Rhode Island Office of State Medical Examiners; Div of Environmental Hazards and Health Effects, National Center for Environmental Health; Div of Toxicology and Human Health Sciences, Agency for Toxic Substances & Disease Registry; EIS officer, CDC

In May 2013, the Rhode Island State Health Laboratories noticed an unusual pattern of toxicology results among 10 overdose deaths of suspected illicit drug users that had occurred during March 7–April 11, 2013. An enzyme-linked immunosorbent assay (ELISA) for fentanyl in blood was positive for fentanyl in all 10 cases, but confirmatory gas chromatography/mass spectrometry (GC/MS) did not detect fentanyl. The mass spectrum was instead consistent with acetyl fentanyl, a fentanyl analog. Acetyl fentanyl, a synthetic opioid, has not been documented in illicit drug use or overdose deaths, and is not available as a prescription drug anywhere. Animal studies suggest that acetyl fentanyl is up to five times more potent than heroin as an analgesic (1).

During May 14–21, 2013, CDC and Rhode Island public health officials conducted a field investigation to determine whether this cluster of 10 deaths represented an increase in the typical number of overdose deaths and what role might have been played by acetyl fentanyl. Data on illicit drug (cocaine, heroin, synthetic cathinones [bath salts], gamma-hydroxybutyric acid, and methamphetamine) overdose deaths during March 1, 2012–March 31, 2013 were abstracted from the Rhode Island Office of State Medical Examiners database and examined using Poisson regression. Data also were abstracted from autopsy reports, toxicology results, and medical records relating to the 10 deaths that were preliminarily positive for acetyl fentanyl. The state health laboratories performed all toxicology testing for acetyl fentanyl.

Investigators found that the number of illicit drug overdose deaths in Rhode Island was significantly higher in March 2013 (21, including 10 attributed to acetyl fentanyl), compared with the monthly average during March 2012–February 2013 (8.9, p<0.001). During the field investigation, two additional acetyl fentanyl overdose deaths were confirmed (dates of death: March 20 and May 16, 2013), bringing the total number of acetyl fentanyl deaths to 12. Among the 12 acetyl fentanyl decedents, ages ranged from 19 to 57 years, and eight were male. All but one of the deaths occurred in northern Rhode Island: six occurred in the same small city and none in the capital city, Providence. Evidence suggested that acetyl fentanyl was administered intravenously in at least four (33%) of the deaths. The route of acetyl fentanyl administration was undetermined for the remaining eight decedents.

The GC/MS toxicology results for 10 of the 12 decedents showed, in addition to acetyl fentanyl, various mixtures of other drugs, including cocaine (58%), other opioids (33%), ethanol (25%), and benzodiazepines (17%). None of the decedents tested positive for fentanyl by GC/MS. Toxicology results for one decedent showed only acetyl fentanyl. Since completion of the field investigation, two persons using acetyl fentanyl together died on May 26, 2013, increasing the number of acetyl fentanyl deaths to 14.

Acetyl fentanyl overdose deaths have recently been confirmed in Pennsylvania (2). If states observe clusters or increases in illicit opioid-related overdoses above expected levels, acetyl fentanyl could be involved and confirmatory testing will be needed. CDC encourages public health officials and laboratories, when feasible, to use an ELISA test to screen specimens from suspected illicit, nonpharmaceutical opioid overdose deaths. If an ELISA test is positive for fentanyl, CDC recommends laboratories conduct confirmatory testing by GC/MS; if no fentanyl is detected by GC/MS, then fentanyl analogs should be suspected, and subsequent testing should be considered.

Naloxone is an opioid antagonist that can reverse potentially fatal opioid-induced respiratory depression and is used as part of the initial treatment of suspected opioid overdose. Because of the increased potency of acetyl fentanyl, larger doses of naloxone might be needed to achieve reversal (3); health-care providers who administer naloxone in emergencies might consider increasing the amount they keep on hand. In addition, expansion of community-based programs that provide opioid-overdose prevention services, including distribution of and training in the use of naloxone, might be an effective strategy to help reduce opioid-related overdose deaths (4).
